# Granular Formation during Apoptosis in *Blastocystis* sp. Exposed to Metronidazole (MTZ)

**DOI:** 10.1371/journal.pone.0155390

**Published:** 2016-07-29

**Authors:** Devi Balkrishnan Dhurga, Kumar Suresh, Tian Chye Tan

**Affiliations:** Department of Parasitology, Faculty of Medicine, University of Malaya, 50603, Kuala Lumpur, Malaysia; Indian Institute of Toxicology Research, INDIA

## Abstract

The role and function of the granular life cycle stage in *Blastocystis* sp, remains uncertain despite suggestions being made that the granules are metabolic, reproductive and lipid in nature. This present study aims to understand granular formation by triggering apoptosis in *Blastocystis* sp. by treating them with metronidazole (MTZ). *Blastocystis* sp.cultures of 4 sub-types namely 1, 2, 3 and 5 when treated with 0.01 and 0.0001 mg/ml of metronidazole (MTZ) respectively showed many of the parasites to be both viable and apoptotic (VA). Treated subtype 3 isolates exhibited the highest number of granular forms i.e. 88% (p<0.001) (0.0001 mg/ml) and 69% (p<0.01) (0.01 mg/ml) respectively at the 72 h in *in vitro* culture compared to other subtypes. These VA forms showed distinct granules using acridine orange (AO) and 4’,6-diamino-2-phenylindole (DAPI) staining with a mean per cell ranging from 5 in ST 5 to as high as 16 in ST 3. These forms showed intact mitochondria in both viable apoptotic (VA) and viable non-apoptotic (VNA) cells with a pattern of accumulation of lipid droplets corresponding to viable cells. Granular VA forms looked ultra-structurally different with prominent presence of mitochondria-like organelle (MLO) and a changed mitochondrial trans-membrane potential with thicker membrane and a highly convoluted inner membrane than the less dense non-viable apoptotic (NVA) cells. This suggests that granular formation during apoptosis is a self-regulatory mechanism to produce higher number of viable cells in response to treatment. This study directs the need to search novel chemotherapeutic approaches by incorporating these findings when developing drugs against the emerging *Blastocystis* sp. infections.

## Introduction

*Blastocystis* sp. is an intestinal parasite with polymorphic forms such as vacuolar, granular, amoeboid[1,2] cystic, avacuolar, and multi-vacuolar forms [3,4] the most commonly detected forms in stools being the vacuolar form. Trophozite and cystic stages are the common forms seen in the life cycle of most protozoans such as *Cryptosporidium parvum*, *Entamoeba histoytica*, *Balantidium coli*[5] and *Giardia lamblia*[6]. The most unusual life cycle stage unique only to *Blastocystis* sp. is the granular form. The description of the life cycles of *Blastocystis* sp. provided previously [1,7] though varied have always incorporated the granular forms as an integral life cycle stage. These forms have been described as vacuolar form of diameter size range between 4 and 15 mm containing granules within the central vacuole. The role of granular forms continues to be in enigma with suggestions that the granules are reproductive, metabolic and lipid [8–10] in nature respectively. Evidence for the lipid granules was provided by Nile blue and Sudan Black B staining [11] which implicated that the granular forms had a storage role. The high numbers of *Blastocystis* sp. seen growing despite the short period in, *in vitro* cultures suggested that the organism must be having another asexual mode of reproduction other than binary fission implicating these granules to be reproductive [2,12,13]. However this was refuted the reason being that the suggested progeny forms showed striking similar characteristics with the metabolic granules described previously as well as the lack of evidence for these to be viable [1,14,15] There was also a postulation that the granules is a result of a mechanism trigger for apoptotic body deposition in the central body in *Blastocystis* sp. cells undergoing programmed cell death [16]. The intracellular granules have been suggested to be heterogeneous and have been described as being myelin-like inclusions, small vesicles, crystalline granules, and lipid droplets [17].

Programmed cell death (PCD), involves the physiological non-inflammatory elimination of damaged or harmful cells during organogenesis or for the proper function of continuous cell-renewal systems in matured organisms [18]. Apoptosis, a common form of PCD, is well characterized by a unique design of morphological alterations in cytoplasm and nucleus and has been investigated in two intestinal protozoans namely *Blastocystis* sp. and *Giardia lamblia*[19]. Several of these features have been described in *Blastocystis* sp. exposed to cytotoxic drug such as metronidazole and surface-reactive cytotoxic monoclonal antibody (1D5)[19–21]. Protozoan programmed cell death or apoptosis is vital in the survival of parasite and its pathogenicity[21]. PCD is used as a defense mechanism for the preservation of cell populations during viral infections, insufficiency of nutrients and other adverse conditions among the unicellular eukaryotes. This is to ensure that some of the cells survive to propagate the genome [22–24]. In the present study by correlating the dynamics of granular formation within the various subtypes of *Blastocystis* sp., to apoptosis caused by treatment with metronidazole, we provide evidence that when a cell is triggered to undergo apoptosis, granular forms play a crucial role in continuing the propagation of *Blastocystis* sp.

## Materials and Methods

### In vitro culture of parasites

Nine isolates of *Blastocystis* sp. comprising of 2 each of subtype 1 (st1), subtype 2 (st2), subtype 5 (st5) and 3 of subtype 3 (st3) were obtained from different symptomatic individuals. Screening protocol to rule out viral, bacterial and protozoan pathogens other than *Blastocystis* sp. were carried out to ensure symptoms in every individual is only related to *Blastocystis* sp. infections which includes bloating stomach, abdominal pain, anal itching and diarrhea. These isolates were maintained in Jones’ medium [horse serum (10%) were added to the medium] through *in vitro* cultivation at 37°C [23,25]. DNA was extracted directly from the culture samples using the QIAGEN Stool Mini Kit (QIAGEN, Hilden, Germany). The isolates were then subjected to polymerase chain reaction (PCR) using seven pairs of sequenced-tagged site (STS) primers, 526 bp for subtype 3 (st3), 351 bp for subtype 1 (st1), and 317 bp for subtype 5 (st5) [26].

This study was approved by the Medical Ethics Committee of the University Malaya Medical Centre (UMMC) Kuala Lumpur, Malaysia according to the Declaration of Helsinki.

### Apoptotic induction via metronidazole

1 x 10^6^ cells ml^-1^ were introduced into a micro- centrifuge tube (Axygen Biosciences, Union City, California, USA) containing the final concentration of 0.0001 mg/ml & 0.01 mg/ml of the drug metronidazole (MTZ), (Discovery Fine Chemicals, Dorset, UK). Tubes with the exact quantity of parasites but untreated were labelled controls. Incubated cells were then harvested every 12 hours up to 96 hours for epifluorescence as well as transmission electron microscopy (TEM) studies.

### Viability of cells

Viability were determined quantitatively via Neubauer hemocytometer chamber (Hausser Scientific, Horsham, PA, USA) and the trypan blue dye exclusion method[27]. Ten μl of the dye were added to the same amount of cells and incubated for 5 min at 30°C. Unstained and stained cells were enumerated as viable and non-viable respectively[25] using the Olympus BX 51epifluorescence microscope, (Olympus, Wetzlar, Germany).

### Cytochemical staining method

#### Detection of apoptotic and late apoptotic forms

Apoptotic cells qualification were carried out using Apoptosis and Necrotic Qualification Kit (Biotium Inc, Hayward, USA). Both treated and untreated cells were washed with PBS. Then, binding buffer (10x), Ethidium homodimer III, Annexin V-FITC (250 μL in TE buffer) and Hoechst 33342 (500 μg/ml in PBS) were added to the cells [27]. Olympus BX 51epifluorescence microscope, (Olympus, Wetzlar, Germany) were used to observe the samples using image analyser software [27]. Bright field images of tryphan blue stained cells were captured using Olympus BX 51microscope and the same parasites stained with FITC were viewed when the filter was changed (BP 450–480). Apoptotic cells were calculated with regard to the percentages of cells which are apoptotic in 100 cells [27].

### MitoCapture^™^Mitochondrial Transmembrane Potential Apoptosis Detection Kit

Harvested cells were centrifuged at 250xg for 10 minutes before re-suspending in diluted MitoCapture reagent. The diluted cells were then incubated in a 5% CO_2_ incubator for 10 minutes. Finally, the cells were centrifuged again at 250xg and the supernatant were discarded before viewing under Olympus BX 51 epifluorescence microscope (Olympus, Wetzlar, Germany) under x400 magnification (incident light transmission). MitoCapture distinguishes healthy and apoptotic cells by detecting changes in mitochondrial transmembrane potential. Image analyser software was used to capture the images.

#### Oil Red O

Cells were washed (2x) with 1ml of PBS (pH 7.4). The cells were further centrifuged (2000*xg*, 10 min) for staining with Oil Red O. 10mg/ml of Oil Red O stock solution were prepared and diluted with sterile distilled water (sdH_2_O) to the final concentration of 2mg/ml. Then, 10μl of 2mg/ml Oil Red O solution were added to the pooled cells before incubation in dark at room temperature for an hour. Finally, the cells were washed with PBS at pH 7.4 and examined under light microscope.

#### Acridine orange staining method to quantify the granular forms

Cells were centrifuged (2000*xg*, 5 min) for acridine orange staining method. 10mg/ml of acridine orange stock solution was prepared and diluted with PBS, pH 7.4, to the final concentration of 0.1mg/ml. A drop of the culture sediment was mixed thoroughly on a clean glass slide with a drop of diluted acridine orange. The cells were examined using epifluorescence microscope Olympus BX 51(Olympus, Wetzlar, Germany) under x400 magnification (incident light transmission). Image analyser software was used to capture the images. Staining intensities were obtained by quantifying percentages of cells stained with acridine orange and the fluorescent intensity in 100 cells. Fluorescent intensity of the cells was expressed as AFU reflected in the following manner: weak (dull green), 1+; medium (green), 2+; flaming red, 3+.

#### DAPI staining method

Cells were washed (2x) with 1ml of PBS (pH 7.4). Then the cells were centrifuged (2000*xg*, 5 min) before staining with DAPI (4’,6-diamino-2-phenylindole) [26].

### Transmission electron microscopy (TEM)

Cells from day 3 culture were washed three times using PBS pH 7.4 and centrifuged at 2000× g, for 5 min. The pelleted cells were re-suspended overnight in 2.5% glutaraldehyde in 0.1 M sodium cacodylate buffer, pH 7.3 at 4°C, washed thoroughly with cacodylate buffer and post-fixed for 30 min in 1% osmium tetroxide in cacodylate buffer. The fixed cells were dehydrated in ascending series of ethanol and embedded in epoxy resin. Semithin sections were stained with toluidine blue. Ultrathin sections were cut using an ultramicrotome, contrasted with uranyl acetate and lead citrate and viewed using a transmission electron microscope[28].

### Statistical Analysis

Statistical analysis was carried out using SPSS Statistics 18.0 software. Correlation test as appropriate was used to determine the correlation value between apoptotic cells and granular cells in every subtypes used in this study. *P*<0.05 was considered statistically significant.

## Results

### Reduction in cell diameter of treated cells

Reduction in cell diameter is one of the hallmark of an apoptotic cell [29]. In this current study, quantification of cell diameter was determined by measuring the diameter of treated and untreated cells. Drug treated *Blastocystis* sp. cells exhibited significant decrease in diameter in comparison to untreated cells (p<0.001) (Table 1). This shows that cell shrinkage does occur in every subtype of *Blastocystis* sp. exposed to metronidazole.

**Table 1:** Diameter (μm) of untreated and treated (0.01 mg/ml and 0.0001 mg/ml) cells of *Blastocystis* sp. after 12 hours culture.

Subtype	Untreated isolates	Treated isolates	
		0.01 mg/ml	0.0001 mg/ml
1	31 ± 7.353	22 ± 3.144^a^	13 ± 5.113^a^
2	32 ± 11.475	20 ± 2.998^a^	15 ± 3.507^a^
3	40 ± 9.836	29 ± 1.177^a^	18 ± 4.337^a^
5	29 ± 7.998	19 ± 2.655^a^	13 ± 4.236^a^

Data are expressed as the mean ± SD.

^a^ p<0.001, comparing the diameter (μm) of untreated cells with treated (0.01 mg/ml and 0.0001 mg/ml) cells

### Apoptotic and viable forms

**Fig 1:**
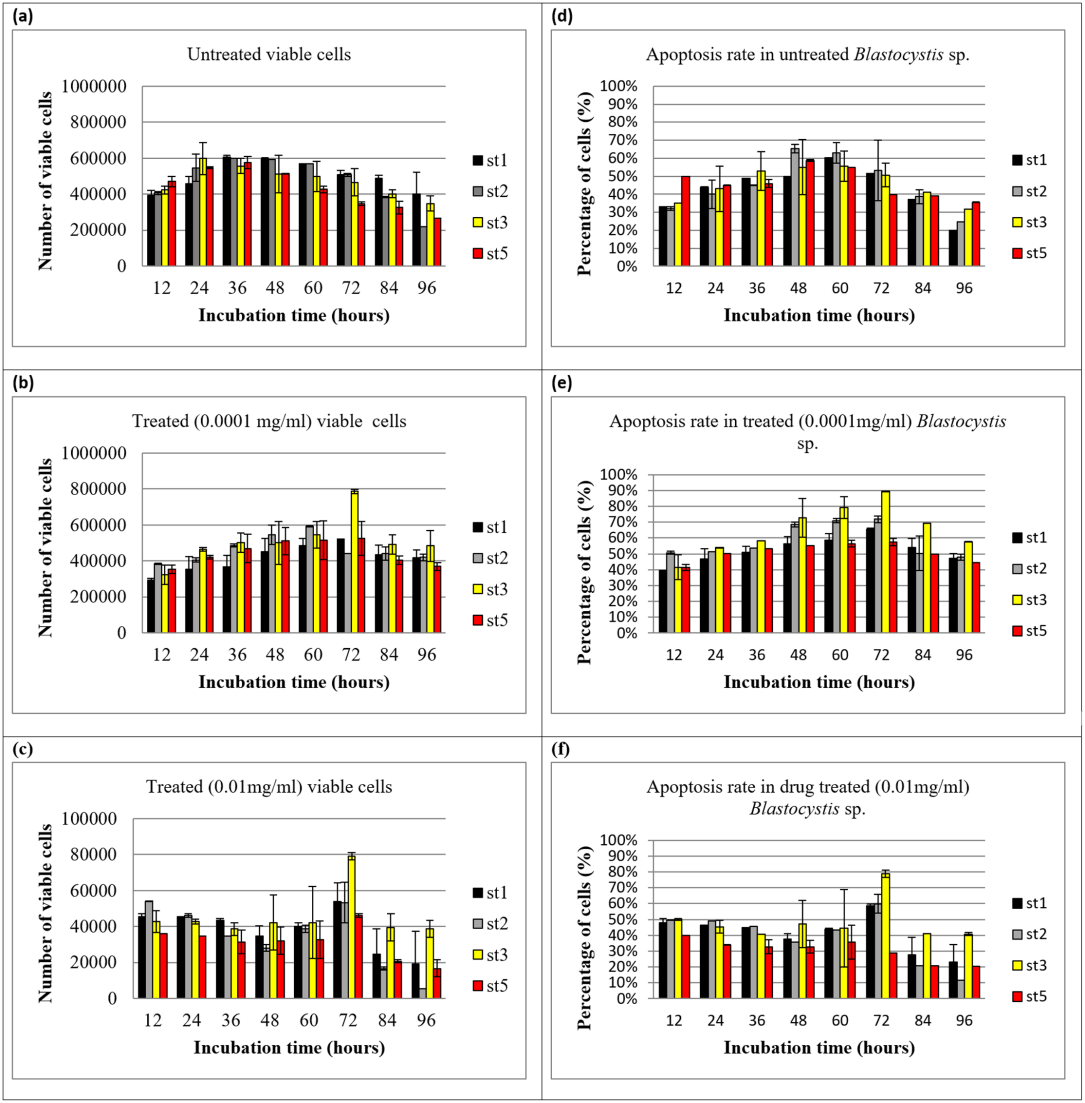
Viable and apoptotic rate of untreated and treated forms of *Blastocystis* sp. (a) viability rate and (d) apoptotic rate of untreated forms of *Blastocystis* sp. of st1, st2, st3 and st5. (b) viability rate and (e) apoptotic rate of treated forms of *Blastocystis* sp.(symptomatic isolates) at 0.0001mg/ml of st1, st2, st3 and st5. (c) viability rate and (f) apoptotic rate of treated forms of *Blastocystis* sp.at 0.01mg/ml of st1, st2, st3 and st5 (st = subtype).

Significant elevation of apoptotic forms were observed at the 72 h culture in subtype 3 in comparison with other subtypes namely subtypes 1, 2, 3 and 5 (Fig 1). In fact, both dosages of drug showed significant elevation p<0.001 and p<0.01 in the apoptosis rate and viable forms of the cells respectively. There was a high correlation between viable and apoptotic cells for subtype 3. The standardization process involved different timing to ensure that the uptake of trypan blue and Annexin V are both correlated well so that the conclusions formed are not biased. Drug treated cells exhibited a good correlation value (r = 0.952) for cells treated with 0.0001mg/ml and (r = 0.909) cells treated with 0.01mg/ml of the drug.

### Apoptotic and non-apoptotic granular forms

**Fig 2:**
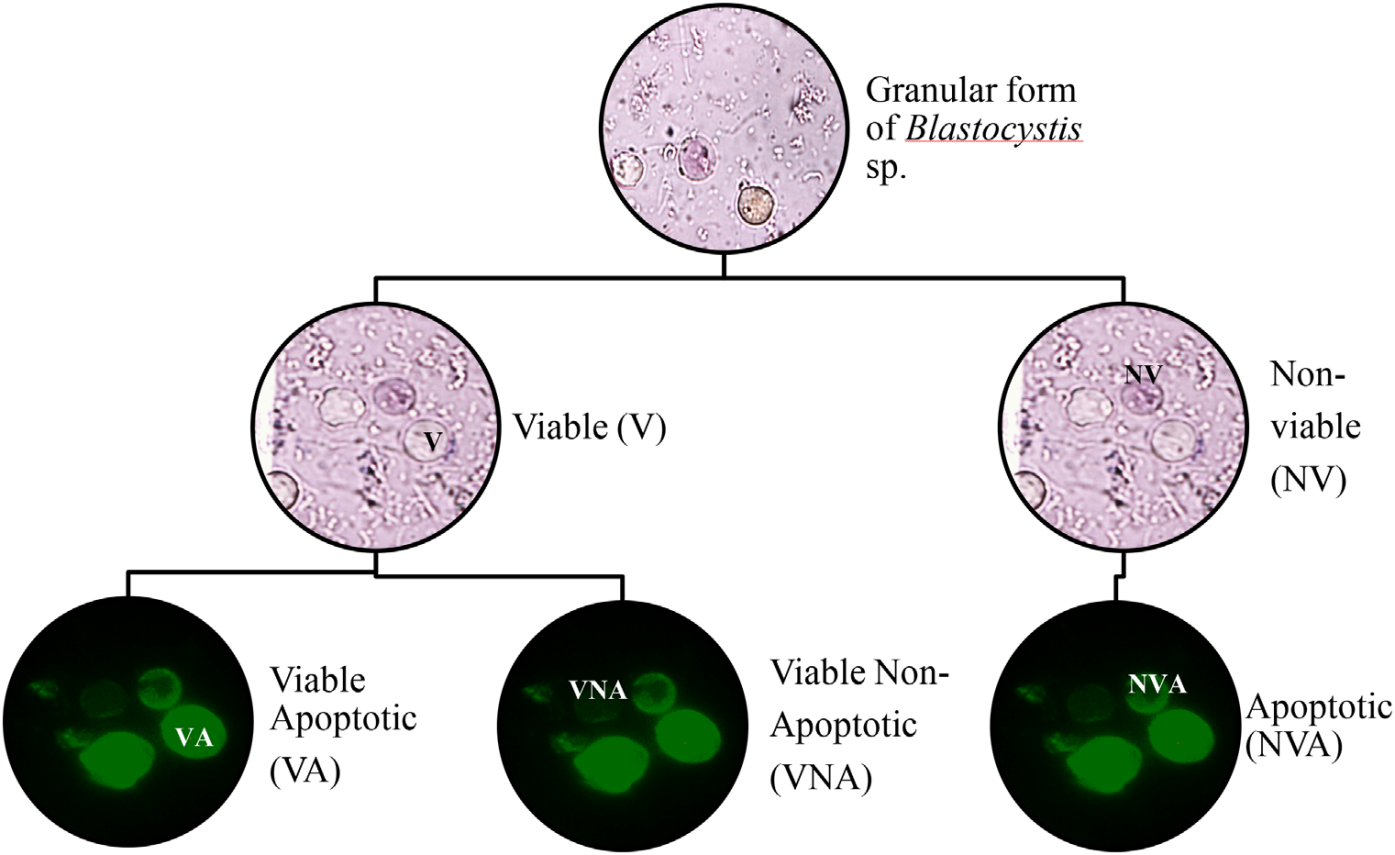
Apoptotic and non-apoptotic granular forms of *Blastocystis* sp. Light microscopy images showing drug treated *Blastocystis* sp. stained with tryphan blue to test the viability and *Blastocystis* sp. stained with Annexin (V)-FITC (green). Annexin V labeled with fluorescein (FITC) identifies apoptotic cells binding to PS exposed on the outer membrane of the cell.

**Fig 3:**
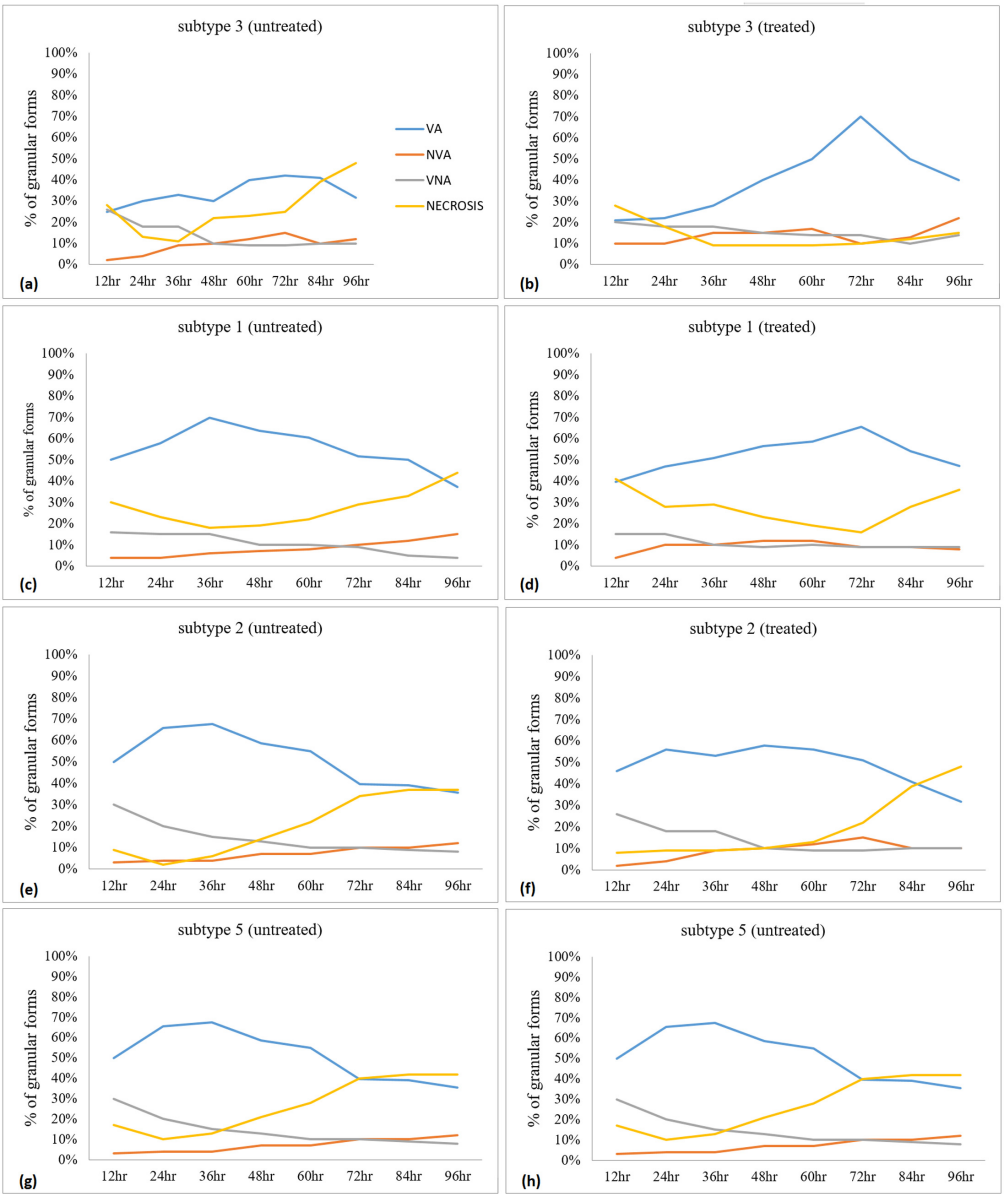
Granular cells of the viable apoptotic (VA) viable non-apoptotic (VNA), non-viable apoptotic ((NVA) forms and necrosis forms. The untreated cells are represented (a) subtype 3, (c) subtype 1 (e) subtype 2 and (g) subtype 5. The treated ones are represented by (b) subtype 3, (d) subtype 1, (f) subtype 2 and (h) subtype 5.

Apoptotic and non-apoptotic granular cells could clearly be differentiated (Fig 2) using FITC Annexin (V). At the same time, the presence of the non–apoptotic granular forms were also noted (Fig 2). When tryphan blue stain were used to distinguish viable and non-viable cells [30], some of the granular cells picked up the dye, while some were left unstained. When observed further, the granular cells which picked up tryphan blue were apoptotic in nature. In the population of granular cells which did not pick up the dye, there were the presence of both apoptotic and non-apoptotic cells. Among the untreated ones, of subtype 3 [Fig 3(a)], subtype 1 [Fig 3(c)], subtype 2 [Fig 3(e)] and subtype 5 [Fig 3(g)], the curves representing apoptotic and non -apoptotic forms were almost similar. Whereas for the treated ones, subtype 3 [Fig 3(b)] showed the highest number of granular cells at the 72^nd^ hour *in vitro* culture in comparison with 3 other subtypes namely subtypes 1[Fig 3(d)], subtypes 2 [Fig 3(f)] and 5 [Fig 3(g)], where at 0.0001mg/ml, 88% (p<0.001) and at 0.01mg/ml, 69% (p<0.01) of cells were granular. The cell populations in each subtype consist of viable non-apoptotic (VNA), viable apoptotic (VA), non-viable apoptotic (NVA) and necrotic cell (N). Vacuolar forms (VNA) were observed in all the treated and untreated cultures at low percentages till 96 hour of culture compared to the initial period when the drug was introduced. The study showed that the drug induced subtype 3 has the highest population of VA cells (70%) at 72 hours, followed by VNA cells (8%), NVA (12%) and necrotic (10%) cells.

### Mitochondrial Transmembrane Potential

**Fig 4:**
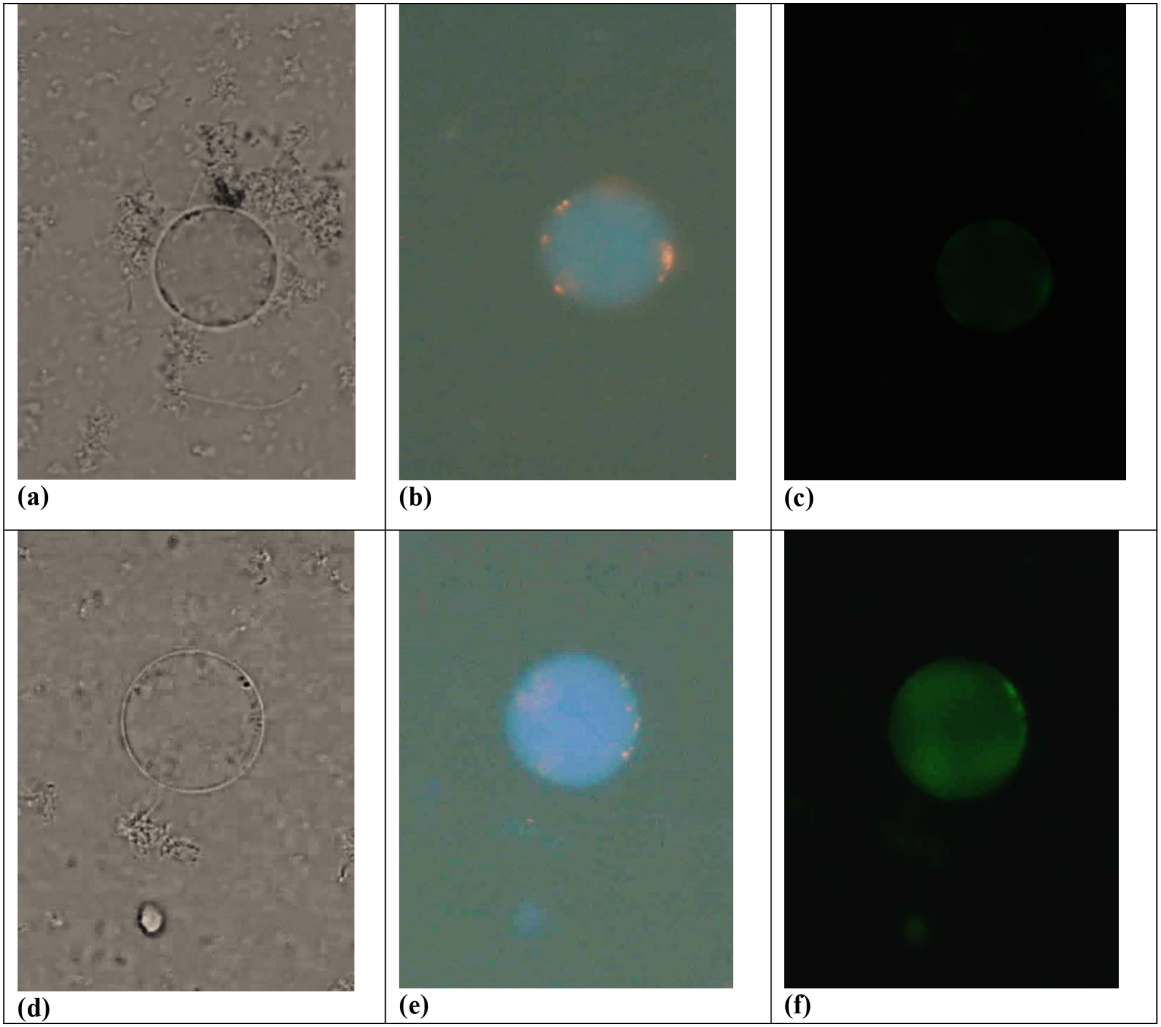
Mitochondrial transmembrane potential. (a) and (d) granular cells viewed under bright field. (b) and (e) MitoCapture accumulates and aggregate in the mitochondria, giving off a bright red fluorescence (c)low intensity of green fluorescence indicating that the granular cells are still viable. (f) high intensity of green fluorescence indicating that the cells are dying (or apoptotic).

In both viable apoptotic (VA) and viable non-apoptotic (VNA) cells [Fig 4(a)], MitoCapture accumulates within the mitochondria, thus emitting red fluorescence signal [Fig 4(b) indicating the presence of intact mitochondria. The intact mitochondria, stained using MitoCapture can be observed in the 70% VA cells and 8% VNA cells of treated subtype 3 at 72 hours. And more intact mitochondria could be observed in both VA and VNA of subtype 3 compared to subtype 1, subtype 2 and subtype 5 as these subtypes have lower number of VA (subtype 1 = 66%, subtype 2 = 41% and subtype 5 = 57%) and VNA (subtype 1 = 9%. Subtype 2 = 9% and subtype 5 = 8%) respectively.

In the 12% non-viable apoptotic (NVA) cells of subtype 3, 9% NVA of subtype 1, 15% NVA of subtype 2 and 11% NVA of subtype 5, the stain appeared to be ‘smeared’ all over the cells, showing that the mitochondria are no longer intact and maybe ruptured.

### Lipid accumulation

**Fig 5:**
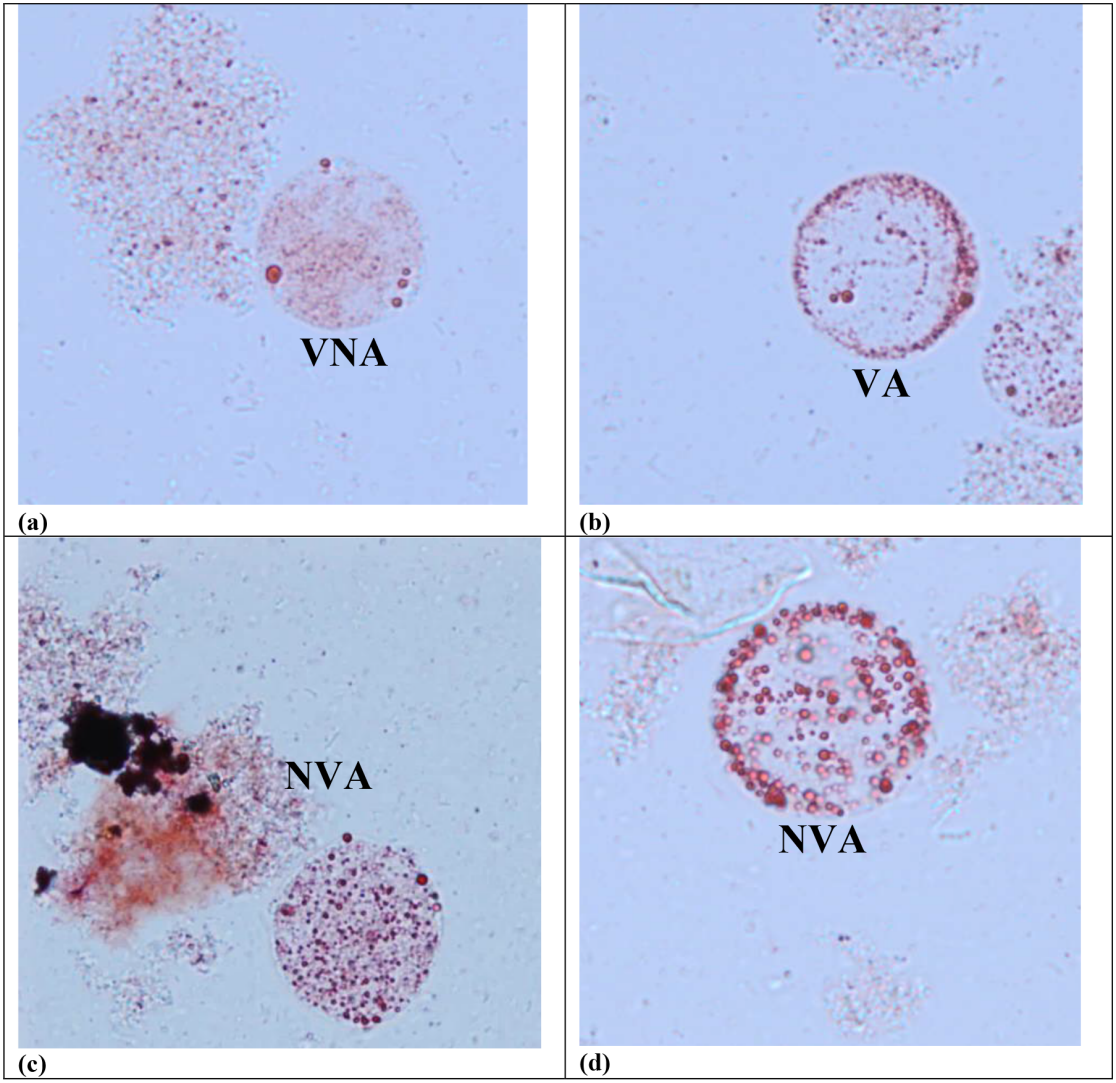
Accumulation of lipid droplets. Presence of lipid in (a) viable non apoptotic (VNA) and (b) viable apoptotic (VA) (c) and (d) non-viable apoptotic (NVA) granular forms of *Blastocystis* sp.

Stored lipids can be used for energy, membrane formation and steroid synthesis. Accumulation of lipid droplets is common in normal cells; but, excessive accumulation of lipid droplets can be an indicator of pathogenesis or metabolic deficiency [31]. (VNA) showed the presence of far less dense lipids [Fig 5(a)]. Viable apoptotic ones (VA) too, show the presence of far less dense lipids scattered to the center of the cell [Fig 5(b)]. The amount of lipid accumulation shows that both VNA and VA are functioning as normal cells. Excessive accumulation of lipids were seen in non-viable apoptotic (NVA) forms due to metabolic deficiency [Fig 5(c) and 5(d)]. This will lead to late apoptotic stage eventually becoming necrotic cells. And when we did quantitative analysis on these cells, subtype 3(70% VA and 8% VNA), subtype 1 (66% VA and 9% VNA), subtype 2 (41% VA and 9% VNA) and subtype 5 (57% VA and 8% VNA) demonstrated the presence of far less dense lipids compared to the 12% NVA cells of subtype 3, 9% NVA of subtype 1, 15% NVA of subtype 2 and 11% NVA of subtype 5 which had excessive accumulation of lipids, at 72 hours.

### Quantification of granules within VA, VNA and NVA forms via acridine orange (AO) and 4’, 6-diamino-2-phenylindole (DAPI)

**Fig 6:**
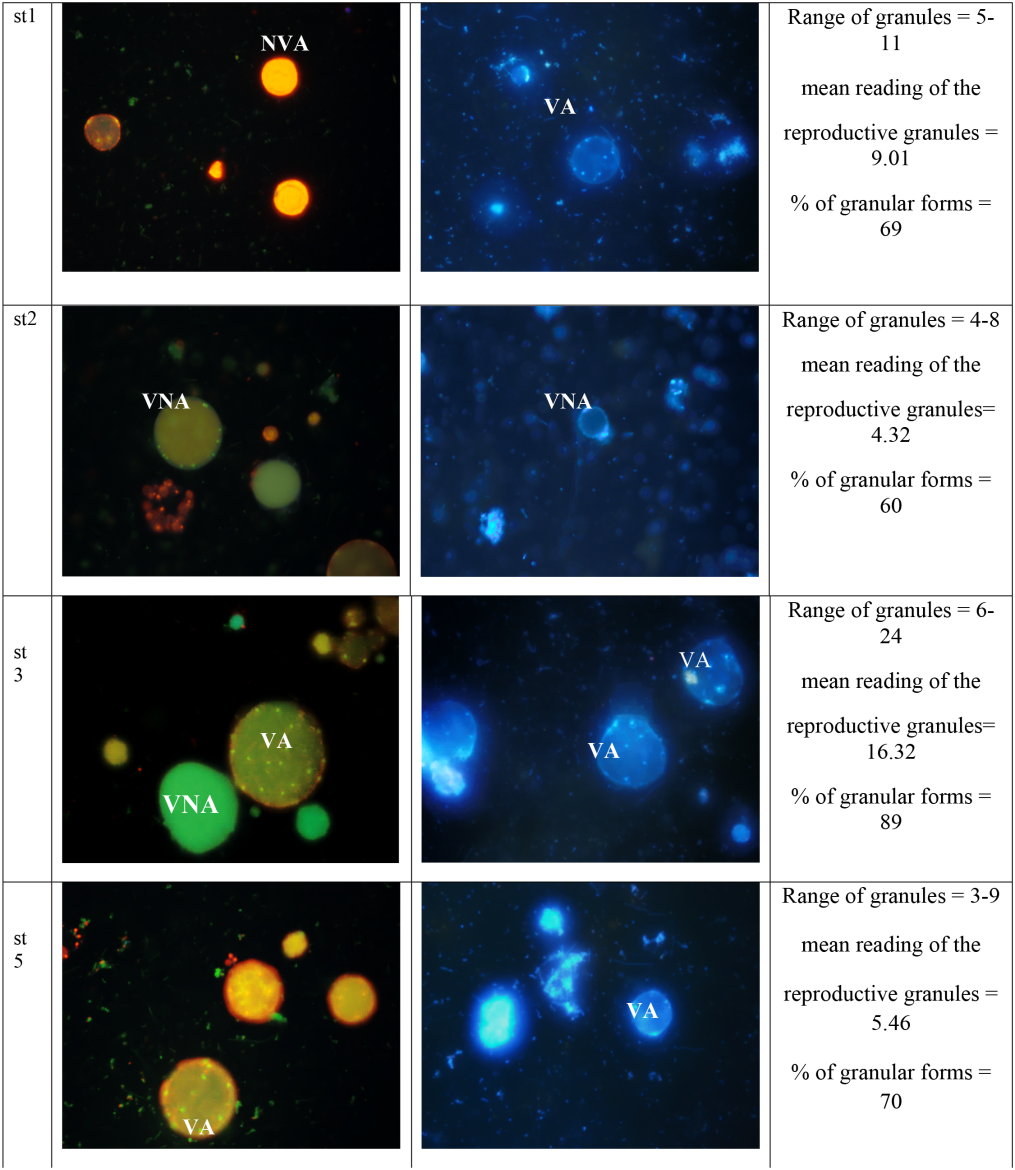
Acridine orange and DAPI stained *Blastocystis* sp. VA = Viable Apoptotic, NVA = Non- Viable Apoptotic, VNA = Viable Non Apoptotic.

**Fig 7:**
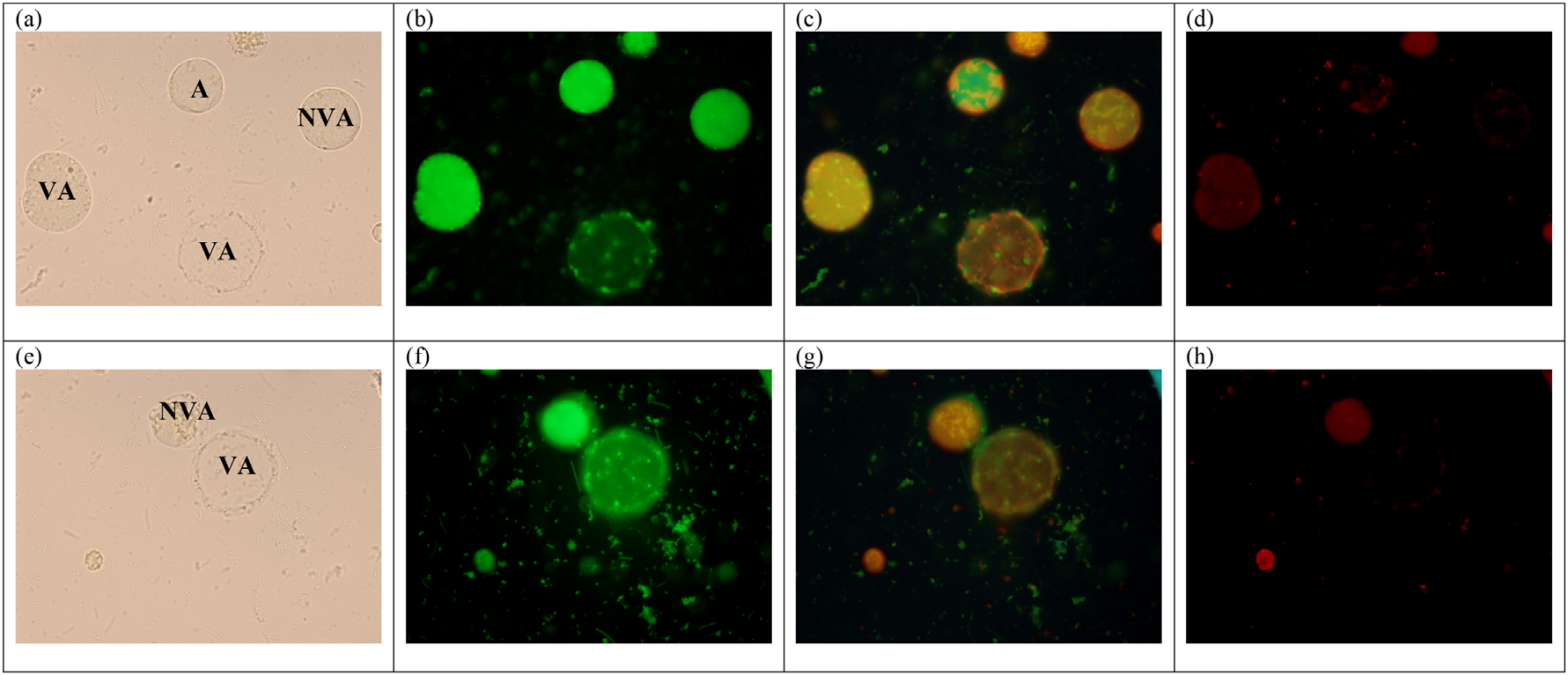
Acridine orange stained granular forms. (a) and (e) light microscopy images showing drug treated *Blastocystis* sp. (b) and (f) *Blastocystis* sp. stained with Annexin (V) (green) (c) and (g) *Blastocystis* sp. stained with acridine orange stain (orange). Annexin V labeled with fluorescein (FITC) identifies apoptotic cells binding to PS exposed on the outer membrane of the cell. Acridine orange stains the DNA of nucleus, mucus, and RNA as bright green, dull green and flaming red–orange, respectively. A = Apoptotic; NA = Non Apoptotic; LA = Late Apoptotic (Necrosis).

**Fig 8:**
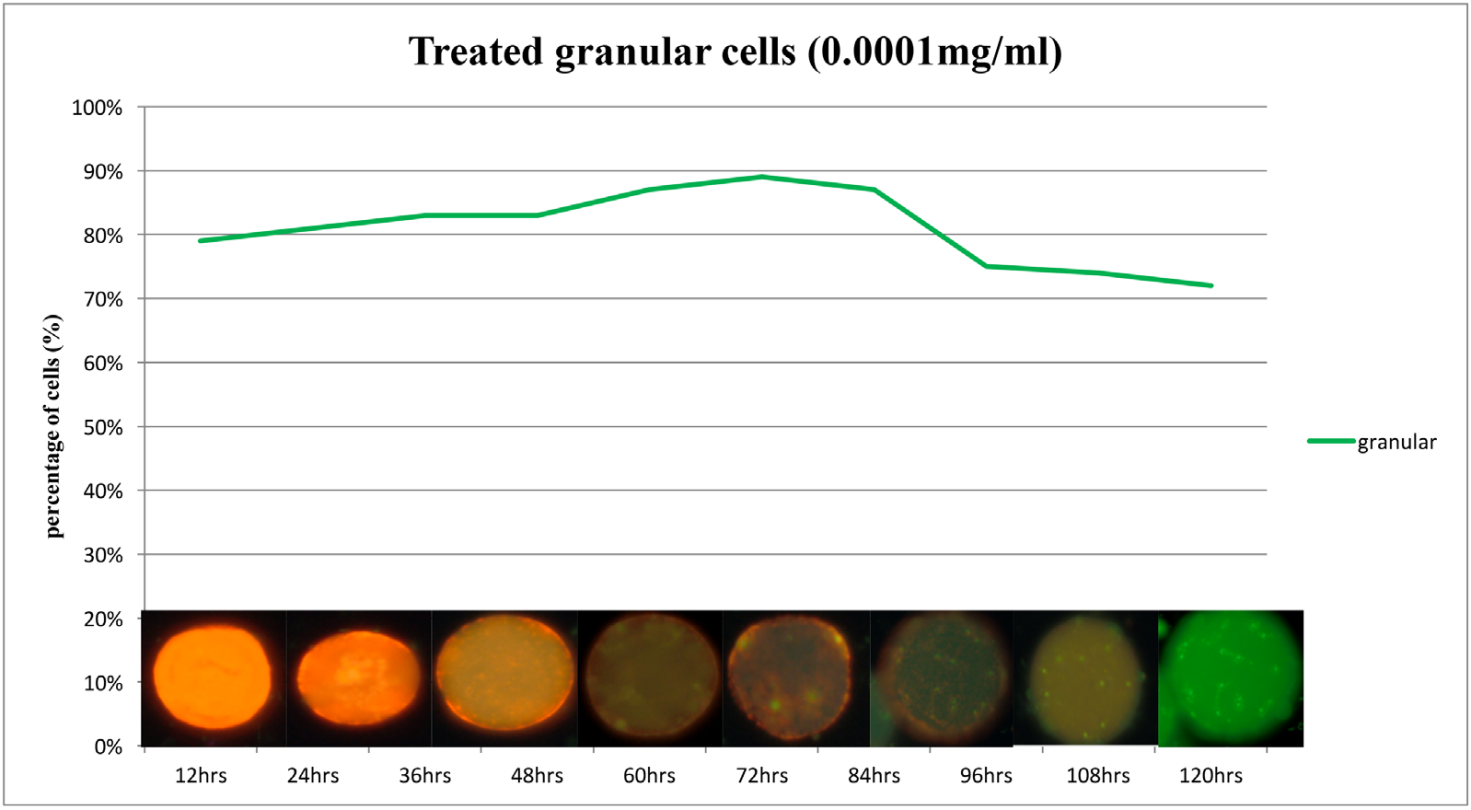
Different shades of acridine orange stained *Blastocystis* sp. *Blastocystis* sp. of subtype 3 (st3) in treated (0.0001mg/ml) condition and images above the graphs show flaming red orange colouration of *Blastocystis* sp. at the 12 hours, decreasing gradually as the hours goes by and clarity of cells start increasing. At the 72 hour to 84 hour, progeny- like forms could be visualized.

Granules were visualized using acridine orange staining (Fig 6) and further were confirmed by DAPI staining. The tabulated mean of granules (Fig 6) show the highest number to be seen in treated subtype 3, 16.32 with VA (70%) at 72 hours, followed by VNA (8%), NVA (12%) and necrotic (10%) cells. For the treated cells subtype 1, mean reading of granules were 9.01 with VA (66%) at 72 hours, followed by 9% VNA cells, 9% NVA cells and 16% necrotic cells. For treated isolates of subtype 2, the mean reading of the granules was 4.32 with VA (41%) at 72 hours, followed by 9% VNA cells, 15% NVA cells and 22% necrotic cells. Meanwhile for the treated subtype 5, mean reading granules was 5.46, with 57% VA cells at 72 hours, followed by 8% VNA cells, 11% NVA and 24% necrotic cells. Acridine orange (AO) stains the DNA of nucleus, mucus, and RNA as bright green, dull green and flaming red–orange, respectively [32] (Fig 7). AO staining provided greater clarity for visualizing the granules for quantification purpose (Fig 8). In the treated subtype 3, almost 70% of cells were granular showing prominent granules. The intense flaming red orange colouration of *Blastocystis* sp. decreased gradually from 30%, especially after the 36^th^ hour (Fig 8). At the 72 to 84 hour, small vacuolar—like forms could be visualized clearly (Fig 8).70% of these forms were viable and apoptotic (VA).8% of cells stained green indicating that these are VNA cells as they were viable and apoptotic evidenced by their respective tests. 22% of cells stained bright orange indicating the presence of RNA. Of these 12% and 10% were NVA and necrotic cells respectively.

### TEM photographs

**Fig 9:**
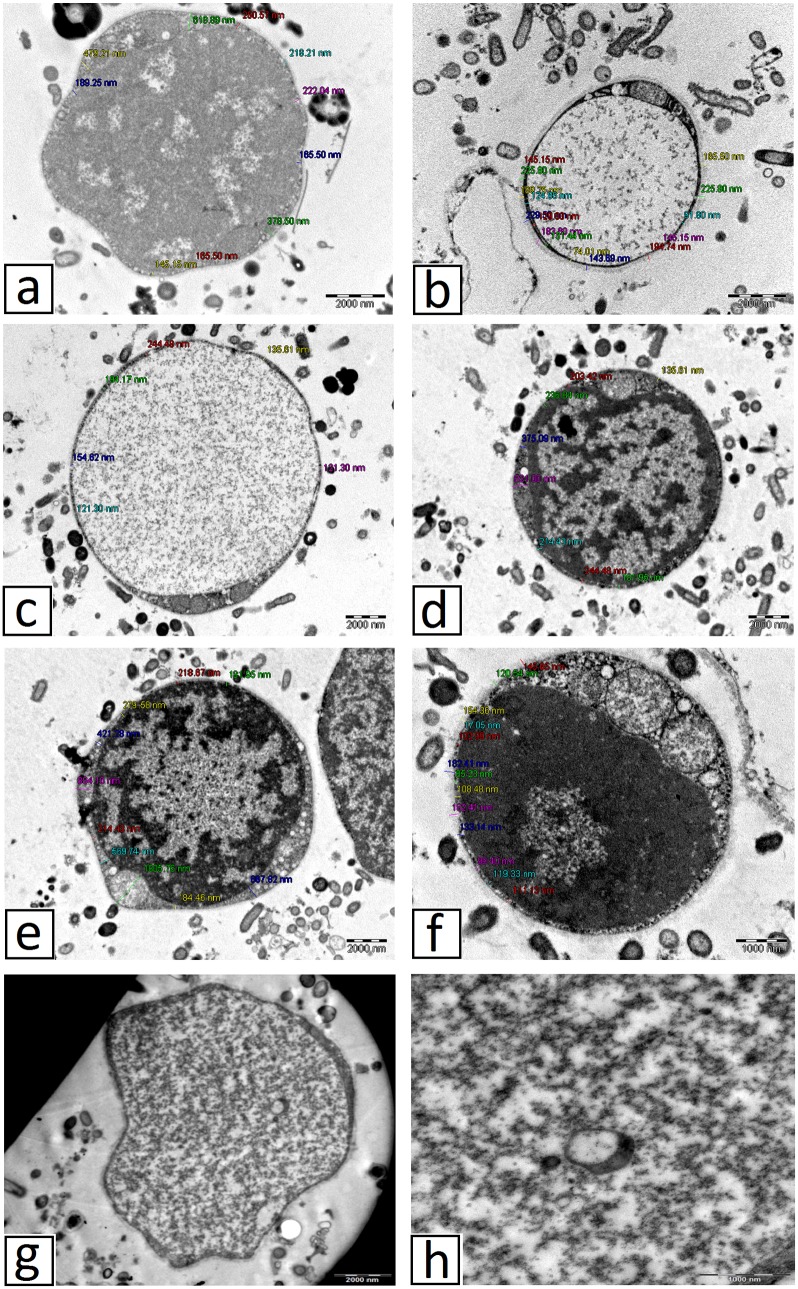
TEM photographs. (a), (c) and (e) shows untreated *Blastocystis* sp. whereas (b), (d) and (f) shows treated *Blastocystis* sp. (g) Electron dense granular forms of treated *Blastocystis* sp. (h) under high magnification; vacuolar form of *Blastocystis* sp. present within the granular forms. **(CR = cristae)**.

Samples after 72 hour of treated cultures were utilized for the TEM procedure. The membranes of the highly electron dense viable apoptotic(VA) granular cells of the parasites appear thicker [Fig 9(d), 9(e) and 9(f)] than the less dense non-viable apoptotic (NVA) cells [Fig 9(a), 9(b) and 9(c)]. The outer membrane of the organelles was fairly smooth but the inner membranes were highly convoluted forming numerous prominent cristae. The MLO showed elongated and branched cristae. The presence of unusual intracellular bodies could not be explained, but this could be the degenerated mitochondria. Irregular shaped electron dense granular forms were viewed in all the VA forms of *Blastocystis* sp. [(Fig 9(g)]. When viewed under high magnification, the granules appeared to be small vacuolar forms of *Blastocystis* sp. with the typical peripheral electron dense nucleus, [Fig 9(h)].

## Discussion and Conclusion

We have shown previously that apoptosis in *Blastocystis sp*. is subtype related [27]. We have also reported that apoptosis in *Blastocystis* sp. occurs via caspase dependent and a smaller percentages of cells undergo caspase independent pathways (using a broad spectrum caspase inhibitor, zVAD.fmk) [33]. Higher percentages of granular cells undergoing caspase like pathway (intrinsic pathway) reported previously [33] prompted us to carry out a more in depth study on the granular apoptotic forms. We extended the observation in the present study and through a series of cytochemical staining and ultrastructural studies demonstrate that granular forms do play a role in apoptosis. This current study highlights the formation of cells which are granular yet viable apoptotic (VA) at the same time. We have also evaluated the morphological features of apoptosis such as, reduction in cell diameter, cell membrane blebbing, and DNA fragmentation before classifying them as apoptotic forms. We have inserted proofs of cell shrinkage in every subtype to subtantiate the findings using annexin V. We have for the first time demonstrated that Viable Apoptotic (VA) forms do exist and postulate that these forms could be the transitional stages to trigger the formation and release of vacuolar forms.

The evidence for this is the close correlation between apoptotic and viable cells in all the subtypes treated with metronidazole. The viable cells did not uptake the tryphan blue dye and at the same time were demonstrated to be apoptotic using the apoptotic staining kit confirming the existence of VA cells (Fig 2). Further evidence for the existence of VA forms has been substantiated from the growth profile, staining and ultrastructural studies carried out in the present study.

In this study, treated cultures had a higher numbers of VA in all subtypes (Fig 3) compared to the controls. The study showed that metronidazole could trigger apoptotic mechanisms [33] which possibly in turn facilitated the formation of granular forms. In other words, when the cells are triggered by certain type of stress (MTZ), some of the vacoular forms undergo apoptosis giving rise to granular form which in turn release the progeny vacoular forms for the purposes of propagation.

In transformational changes during excystation [34] and encystation [13] mitochondria have showed variation in shape which is an indicator that energy is involved during these biological changes. Hence during apoptosis similar cellular changes could be taking place and therefore we decided to include mitochondria into our study. The study therefore attempted to use Mito Capture solution to assess if transmembrane potentials could have a change. The bright red fluorescence exhibited while using the MitoCapture dye indicates the presence of intact MLO within the cell environment In non-viable apoptotic (NVA) cells, MitoCapture remains within the cytoplasm of the cell [Fig 4(f)]. It can be postulated that the drug would have triggered the mitochondria membrane to be more permeable thus resulting in the cytochrome c (Cyt C) release into the cytosol, triggering the downstream events in the apoptotic mechanism. The present finding should have been better substantiated to assess the status of mitochondrial cytochrome C release into the cytosol. However this we feel should be the basis of our future work. Nevertheless, the current findings with regard to the VA granular forms could be easily correlated to the depolarization of the mitochondrial membrane potential as we have previously reported that stress induced *Blastocystis* sp. cell undergo both caspase dependent and independent pathways and mitochondria acts as a central station for both pathways.

These VA granular forms when stained with Oil Red O exhibited a light red staining intensity and the lipid droplets were scattered towards the center of the cells. Minute lipid droplets were found at the peripheral of the VNA forms. Lipid droplet accumulation is usually an indicator whether a cell is functioning normally [35]. But the NVA cells exhibited high red staining intensity suggestive of a high accumulation of lipids, an evidence of metabolic deficiency which substantiated the fact that granular non-viable apoptotic cells will eventually succumb to cell death. This staining again shows that granular forms exist in two variant forms. The NVA ones will succumb to death but the VA forms will release the smaller vacuolar ones.

Vacuolar forms when stained with DAPI exhibited blue stained nucleus in the periphery but the granular forms exhibited blue stained granules within the cell body. It is obvious that the granules contain the nuclear content. Acridine orange staining further supports this with the evidence of granules showing bright green fluorescence, an indication of the presence of DNA.

During the initial period upon drug induction, the organisms showed red orange colouration when stained with acridine orange (AO). This could be due to massive cellular re-organization occurring due to the initiation of the apoptotic activity. The intense colouration began to decrease providing greater clarity revealing prominent granules within cell body (Fig 8).

The presence of vacuolar forms or the VNA forms imply that these vacuoles may have developed from the released granules Our resultsanother study that reported [36]. Our results therefore concur with another study that reported that the structure was not a true vacuole but the site of development of granules during transition to granular form [36].

The intra-variation in terms of number of granules within the subtypes also clearly shows that granular formation is influenced by subtypes. We have previously shown that apoptosis is subtype influenced [27]. The present finding implicates that there appears to be a relationship between apoptosis and granular formation. Subtype 3 has been shown to have the highest apoptotic rate and in the present study the same subtype showed the highest number of mean granules per cell which is 16.32 compared to 9.01 in subtype 1 and 5.46 in subtype 5.

Further evidence comes from the transmission electron microscopy (TEM) studies which exhibited the presence of small vacuolar forms of *Blastocystis* sp. within the electron dense granular form of treated *Blastocystis* sp. A distinct thick membrane surrounds the highly dense viable apoptotic (VA) granular forms probably to cushion the highly densed granules before being released into the cell environment. This suggests that the thickness of membrane do play a role in granular formation as denser the granules, the thicker the membranes. High numbers of membrane-bound organelles or mitochondria-like organelles (MLO) were observed in the viable apoptotic (VA) *Blastocystis* sp. in comparison with the non-viable apoptotic (NVA) *Blastocystis* sp.

Binary fission has been reported as the sole method of reproduction for *Blastocystis* sp. A group of researchesnother has described crystalline granules, small vesicles and lipid droplets in the central vacuole of the granular form [1]. Plasmotomy, endodyogeny, schizogony, and sporulation although suggested have not been supported by strong evidence. A group of researches, proposed that there are two types of granules [1]. One type was thought to develop into *Blastocystis* sp. cells and another, located at the periphery of the cell to play a role in the cell metabolism. There is also a proposal of the existence of three types of granules exist i.e. metabolic, lipid and reproductive granules respectively. Metabolic granules reported to be existing in the cytoplasmic region, lipid granules in the cytoplasm and central vacuole and the reproductive granules in the central vacuole [37]. These observations have not been confirmed, and another study has described crystalline granules, small vesicles and lipid droplets in the central vacuole of the granular form [17]. Lipid droplets were also noted in the cytoplasm [17]. Granules that appear similar to those in the central vacuole were found in small vacuoles and vesicles in the cytoplasm of granular cells [38]. Central vacuole has been reported to function in schizogony and endodyogeny via the development of reproductive granules [39,40]. This has not been supported by further studies.

Altered cell conditions have been reported to increase the granular cells [17,37,41,42]. This is probably based on the observations from the present study that apoptosis could trigger granular formation. The cytochemical staining and the TEM images prove that there is another mode of asexual reproduction taking place within the *Blastocystis* sp. besides binary fission. This concurs with another finding which showed that the treated cells could have reverted to granular forms and the treatment enhanced the reproductive potential of the parasite and accounts for the possible release of the reproductive granules thus giving rise to the number of parasites in cultures [43].

Cell suicide mechanisms have already been demonstrated in prokaryotic and eukaryotic cells [44]. Apoptosis may not serve as a mechanism only for programmed death but to increase the viable cells thus enhancing their pathogenic potential. In other words, when the cells are triggered by certain type of stress, the population of cells prepare themselves to make sure that their species continue to propagate. In unicellular organisms population, programmed cell death chooses the fittest cell and regulates the process of cell differentiation and cell cycle of the whole population [45]. Stramenophiles (known as heterokonts) include unicellular and multicellular protists, example, water molds, slime nets, chrysophytes, diatoms, brown algae and the recently classified *Blastocystis* sp. Zonoria (zonoria antheridia and zonoriaoogonia) are among the representative genera of phaeophyta or brown algae which are general features of heterokonts. The oogonia of zonoriaoogonia were reported to contain single large egg with granular cytoplasm [46]. This further strengthens the finding from this study that the granules found within the granular forms are actually the reproductive granules.

Metronidazole is still a popular choice of drug in the treatment of infections caused by protozoans. Previously, a group of researchers reported that metronidazole induced carcinogenicity in rodentsrodents [47]. Several studies in the past have clearly demonstrated the resistance of *Blastocystis* sp. isolates against metronidazole [8,43,48,49]. In another study, nine out of 21 individuals (symptomatic) were positive for *Blastocystis* sp. and of these; six were infected with *Blastocystis* sp. subtype 3.subtype 3[50]. Subtype 3 was also reported to be subtype with pathogenic potential [30]. In the same study, a vast majority of the patients reported failure with metronidazole. Findings in the present *in* vitro study provide a substantiating basis for the difference in treatment response seen in *Blastocystis* sp. infected patients when treated with metronidazole. We have previously established that subtype 3 may possess higher sensitivity to metronidazole as isolates of this subtype exhibits high fluorescence intensity for caspase-like proteases [33]. Whether or not, blocking of the caspases (initiator and or executioner caspases) have any modulatory effect towards the apoptosis mechanism itself should be assessed in future. The *in vitro* findings reported in this current study, substantiates the reason why metronidazole (MTZ) affects differently among patients. Recent findings have also reported on the ineffectiveness of duotheraphy (trimethoprim/sulfamethoxazole) at inhibiting *Blastocystis* sp. Anothergrowth in axenic cultures [51]. Another study, has reported that metronidazole could be the trigger point in enhancing the reproductive potential of the parasite [43]. The present study for the purposes of continuity and comparison have confined the drug used to be metronidazole. The study confined only to metronidazole and in future more studies need to be carried out using other potential drugs to see if the consequence on apoptosis is the same. The implications of this study is that metronidazole has been shown to exert different effects based on the variations of apoptosis rate in every subtype of *Blastocystis* sp. and this implies that it is necessary to ascertain the subtype of *Blastocystis* sp. before prescribing the treatment. The formation of VA forms in *Blastocystis* sp. could be the trigger to release many progeny vacuolar forms especially if patient stops treatment midway or show no compliance. This can lead to complications. Hence, this study has important contribution when developing drugs against *Blastocystis* sp. infections by targeting the various apoptotic pathways.

The present findings incorporating apoptosis and involving a series of staining techniques with ultrastructural and growth profile studies have shown the existence of VA granular forms which provides a greater clarity towards their role and function in the life cycle of *Blastocytis* sp. Clarity now has been conferred on the role of the enigmatic granular form and the respective mechanism involved in *Blastocystis* sp. undergoing apoptosis. Our study for the first time has shown that in *Blastocystis* sp., when cells undergo programmed cell death, new cells re-emerge to survive and continue propagating. Apoptosis is crucial as a contributory mechanism towards understanding the pathogenesis of the organism. The mechanism need to be explored at the molecular levels and possibly extend to become a model to study better cell multiplication. *Blastocystis* sp. proves to be an excellent model to understand the apoptosis mechanisms due to the existence of the different rate of apoptosis in the various subtypes of *Blastocystis* sp. This thus enable us understand the apoptosis mechanism of unicellular protozoans better.
